# Supplementary material for: The Floral Signals of the Inconspicuous Orchid Malaxis monophyllos: How to Lure Small Pollinators in an Abundant Environment
Source: Biology (Basel). 2022 Apr 21;11(5):640. doi: 10.3390/biology11050640 (PMC9137910; doi:10.3390/biology11050640)
Supplement: Supplementary file 1 [file biology-11-00640-s001.zip › biology-1638771-supplementary.pdf]

biology

MDPI

Supplementary Materials

The Floral Signals of the Inconspicuous Orchid *Malaxis monophyllos*: How to Lure Small Pollinators in an Abundant Environment

Edyta Jermakowicz, Joanna Leśniewska, Marcin Stocki, Aleksandra M. Naczk, Agata Kostro-Ambroziak and Artur Pliszko

Table S1. Relative chemical composition (% of TIC) of volatile organic compounds emitted by *Malaxis monophyllos* flowers.

Compound	% of TIC	
	I	II
TERPENES		
**monoterpenes**	**0,74**	**2,52**
β-Pinene	trace	0,89
δ-3-Carene	0,56	0,84
Limonene	0,18	0,78
**sesquiterpenes**	**26,87**	**11,99**
α-Longipinene	trace	0,12
a-Copaene	0,15	0,27
β-Elemene	2,43	0,42
Cyperene	1,41	1,31
β-Caryophyllene	0,75	0,21
α-Guaiene	0,72	0,24
α-Humulene	0,78	0,17
Rotundene	0,11	0,13
Sesquiterpene C15H24	0,23	0,23
γ-Gurjunene	0,35	0,47
β-Selinene	8,45	2,09
(Z,E)-α-Farnesene	7,16	0,40
α-Selinene	trace	0,18
(E,E)-α-Farnesene	1,21	4,59
7-epi-α-Selinene	0,43	0,31
δ-Guaiene	0,36	0,58
Sesquiterpene C15H24	1,93	0,26
β-Atlantol	0,08	trace
Sesquiterpene C15H24	0,31	trace
**diterpenes**	**0,98**	**1,02**
(E,E)-7,11,15-Trimethyl-3-methylene-hexadeca-1,6,10,14-tetraene	trace	0,23
(E,E)-Geranyl linalool	0,98	0,79
AROMATIC COMPOUNDS		
**aromatic aldehydes**	**0,62**	**0,51**
Benzaldehyde	0,62	0,51
**aromatic alcohols**	**0,70**	**0,74**
Benzyl alcohol	0,20	0,50

Eugenol	0,50	0,23
**ALIPHATIC COMPOUNDS**		
**aliphatic esters**	**0,29**	**0,16**
Methyl 2-oxononanoate	0,29	0,16
**aliphatic acids**	**trace**	**0,25**
Heptanoic acid	trace	0,25
**aliphatic ketones**	**2,41**	**6,36**
2-Methyl-3-heptanone	1,13	4,65
2-Nonanone	0,56	1,01
3-Decanone	0,45	0,31
4-(2-Hydroxy-2,6,6-trimethylcyclohexyl)-3-buten-2-one	0,27	0,39
**aliphatic aldehydes**	**28,77**	**27,74**
Heptanal	2,34	2,26
Octanal	5,18	3,36
(E)-2-Octenal	0,31	0,18
Nonanal	1,02	1,87
(E)-2-Nonenal	0,59	0,71
Decanal	0,41	0,50
(E)-2-Decenal	0,38	0,16
Undecanal	0,13	0,15
(E)-2-Undecenal	0,33	0,13
Dodecanal	0,10	0,34
(Z)-7-Tetradecenal	0,20	0,15
Tetradecanal	0,22	0,19
(Z,Z,Z)-7,10,13-Hexadecatrienal	0,30	0,12
Heptadecatrienal	0,30	0,93
Octadecatrienal, isomer 1	6,87	1,26
Octadecatrienal, isomer 2	6,31	2,23
Octadecatrienal, isomer 3	2,79	9,62
Octadecatrienal, isomer 4	0,99	3,58
**aliphatic alcohols**	**17,82**	**20,61**
2,3-Butandiol	4,28	2,05
(Z)-3-Hexen-1-ol	0,11	0,04
1-Hexanol	0,86	1,31
2-Methyl-3-heptanol	1,38	2,93
1-Heptanol	2,02	3,40
1-Octen-3-ol	0,65	1,08
3-Octanol	0,92	1,25
(E)-2-Octen-1-ol	0,10	trace
1-Octanol	3,57	3,58
5-Nonanol	0,16	0,28
2-Nonanol	trace	0,23
2,5-Dimethyl-3,4-hexanediol	trace	0,44
(Z)-3-Nonen-1-ol	0,93	1,41
(E,Z)-3,6-Nonadien-1-ol	0,15	0,21
(E)-2-Nonenol	trace	0,29
1-Nonanol	0,77	0,88
1-Decen-3-ol	1,06	0,70
3-Decanol	0,26	0,31

(E)-2-Decen-1-ol	0,36	0,15
1-Decanol	0,22	0,08
**alkanes**	**20,51**	**27,96**
n-Tridecane	0,10	0,09
n-Pentadecane	1,62	0,84
n-Heptadecane	0,11	0,14
n-Nonadecane	0,32	0,38
n-Eicosane	1,11	1,51
n-Heneicosane	14,41	19,80
n-Docosane	0,83	1,52
n-Tricosane	2,01	3,67
**other aliphatic compounds**	**0,28**	**0,14**
2-Heptylfuran	0,14	trace
Dihydroedulan	trace	0,06
1-Heneicosene	0,14	0,08
